# Research progress on multisensory stimulation therapy for pain during retinopathy of prematurity screening

**DOI:** 10.3389/fped.2025.1623188

**Published:** 2025-08-20

**Authors:** Chunlan Tao, Chuan He, Jinghua Tang, Yongli Li, Yuqin Chen

**Affiliations:** ^1^ Department of Neonatology, Affiliated Hospital of North Sichuan Medical College, Nanchong, Sichuan, China; ^2^ Department of Emergency, Affiliated Hospital of North Sichuan Medical College, Nanchong, Sichuan, China; ^3^ Department of Nursing, Affiliated Hospital of North Sichuan Medical College, Nanchong, Sichuan, China

**Keywords:** preterm infants, retinopathy of prematurity, multisensory stimulation, mass screening, pain

## Abstract

Retinopathy of prematurity (ROP) is a retinal disease characterized by abnormal vascular proliferation, primarily associated with premature delivery and low birth weight. Advances in perinatal and neonatal care have increased survival rates but have also contributed to a rising incidence of ROP, necessitating regular ROP screening. However, the screening procedure, which involves an eyelid speculum and ophthalmoscope, frequently induces pain. Given the potential adverse effects of pharmacological interventions, non-pharmacological pain management is essential. Multisensory stimulation (MSS), integrating auditory, olfactory, gustatory, and tactile stimuli, is recommended by guidelines due to its favorable safety profile and proven analgesic efficacy. This review synthesizes evidence regarding MSS for pain management during ROP screening, focusing on three aspects: operational definitions of MSS [tactile stimuli like non-nutritive sucking (NNS), auditory interventions such as white noise, and gustatory agents including 24% sucrose]. Analgesic mechanisms, particularly sensory pathway competition and endorphin release; and clinical efficacy, with meta-analyses demonstrating superior outcomes of combined interventions (white noise combined with NNS) compared to single-modal approaches. This review aims to provide evidence-based guidance for effectively implementing MSS strategies, ultimately minimizing procedural pain.

## 1 Introduction

ROP is one of the leading causes of visual impairment in children worldwide (1). In recent years, with an increasing proportion of advanced maternal age and improved neonatal care in China, the number of preterm births and incidence of ROP have progressively increased. Current data indicate the incidence of ROP in China is 15%**–**20%, with a particularly high incidence (61.8%) among extremely preterm infants born at 23–28 weeks’ gestation (2). However, ROP is preventable in most cases, and regular ROP screening for high-risk preterm infants is an effective method to prevent disease progression (3). Nevertheless, the use of mydriatic agents and eyelid speculums during screening procedures can cause moderate to severe pain in neonates (4). These painful stimuli induce acute physiological and behavioral responses, including irritability and crying, which lead to increased heart rate, reduced blood oxygen saturation, compromised fundus image quality, difficulty in clinical interpretation, and potential long-term neurodevelopmental sequelae, such as stress disorders, attention deficits, and learning impairments (5). Although clinical studies have demonstrated that topical procaine anesthesia reduces procedural pain (6–8), neonates still exhibit intense distress behaviors (3). Pharmacological analgesia also carries potential risks, including hepatotoxicity and anaphylactic reactions. Therefore, non-pharmacological interventions have gradually become the preferred clinical approach. Multimodal approaches tend to be more effective than single interventions, and MSS is particularly effective in reducing pain (8). Previous research indicates that MSS enhances maternal-infant attachment, improves feeding efficiency, and promotes brain function and neural development (9–11). Currently, interventions to manage procedural pain during ROP screening vary considerably. This review synthesizes the theoretical basis, mechanisms, and clinical efficacy of MSS to provide a reference for its application in pain management during ROP screening of preterm infants.

## 2 Theoretical basis of MSS

### 2.1 Origin and development of MSS

In the 1970s, Dutch scholars proposed the concept of MSS. By providing gentle multimodal stimuli (such as visual, auditory, tactile) within a specially designed spatial environment, this therapy helps patients release emotional stress, activates positive affective memories, restores sensory equilibrium, and promotes psychological stability. Initially, MSS targeted children with autism spectrum disorder, using structured sensory input to improve patients’ perception and behavioral abnormalities. The concept was subsequently introduced to the United Kingdom, adapted, and renamed “Multi-Sensory Environment (MSE).” Clinical applications gradually expanded to Alzheimer's disease, cognitive impairment, and pediatric behavioral disorders, establishing MSS as a valuable neurological rehabilitation intervention. In 2001, based on pain regulation theories, the team led by Italian scholar Professor Bellieni proposed the “3t sensory saturation method (3tSS).” This method uses three core stimuli (taste, touch, and verbal interaction), supplemented by olfactory, auditory, and visual inputs, competing with pain signals for neural pathways, thus suppressing pain perception (12). This innovative theory has significantly advanced the application of MSS in neonatology, particularly in managing procedural pain among preterm infants.

### 2.2 Operational definition of MSS

MSS refers to providing two or more sensory stimuli, including sight, hearing, smell, touch, and taste (13). Due to the immaturity of postnatal visual development, few related studies exist, while olfactory interventions require substantial habituation time. Consequently, tactile, auditory, and gustatory interventions are widely used to alleviate pain during ROP screening. Auditory interventions include white noise and music therapy; tactile interventions include kangaroo care and touch; taste interventions involve sweeteners and breast milk. Although MSS effectively alleviates pain during ROP screening, the sensory intervention methods, initiation timing, and durations differ among studies. For instance, Hou et al. administered white noise 1 h before screening (3), whereas Ren et al. began white noise stimulation 1 min prior to screening (14). Fu et al. recently analyzed auditory interventions for neonatal procedural pain. They found music therapy, averaging 56.7 dB, initiated 10–15 min before the procedure and continuing until 5–10 min afterward. Parental voice interventions (∼50 dB) begin 10 min prior and last until 10 min afterward or until the infant calms down. White noise (45–55 dB) starts 1 min before and ends 3 min after the procedure (15). However, the optimal timing and duration of sensory interventions remain uncertain. Although most researchers use 25% glucose for gustatory interventions, other studies use 10% glucose, 33% glucose, or 24% sucrose. The analgesic effects of different sweetener concentrations and dosages remain unclear.

### 2.3 Application of MSS in neonatal procedural pain relief

Pain is an unpleasant emotional experience associated with actual or potential tissue damage and involves complex physiological and psychological activities (16). Currently, pain is regarded as the fifth vital sign (17). However, preterm infants have immature organ systems and inevitably undergo frequent painful procedures (18), experiencing pain more intensely than full-term infants. Wang et al. reported that neonates experience, on average, more than five painful procedures daily during hospitalization, with a neonatal pain incidence of 83.63% (19). To address this challenge, researchers have explored non-pharmacological analgesic strategies. Studies examining MSS application during acupuncture found MSS effectively reduced pain without adverse reactions (12, 20). MSS provides multiple sensory inputs, activates unimodal sensory and related cerebral regions, regulates various biological mechanisms, enhances cerebral cortex activity, and leverages sensory strengths to compensate for deficits in vision and touch (21).

## 3 Mechanism of MSS action in ROP screening pain

In 1965, Wall and Melzack proposed the gate control theory, suggesting that the brain functions as a filter blocking certain stimuli. In 2001, Professor Bellieni suggested that various sensory stimuli compete with pain signals, blocking pain pathways to the cerebral cortex and thus reducing pain transmission (22). Given the closed management model in China's neonatal intensive care units, parental participation, such as kangaroo care and skin-to-skin contact, is often limited. Consequently, clinical protocols frequently utilize maternal voice and breast milk interventions.

### 3.1 Mechanisms of tactile intervention

Tactile intervention alleviates neonatal pain by activating sensory pathways that compete with nociceptive signals. Currently, tactile interventions used in MSS include kangaroo care, touching, and NNS.

Kangaroo care involves placing the newborn upright at a 60-degree angle on the chest of the mother or another relative. This promotes cutaneous, vestibular, and motor stimulation, enhances thermoregulation, activates the vagus nerve signaling pathway, and inhibits the release of pain-related neurotransmitters, thereby reducing neonatal pain responses (23). Touching involves the scientific and skillful stimulation of the newborn's skin and body, which activates cutaneous receptors, transmits signals to the central nervous system, and triggers vagal excitation to counteract pain and injury (24). Several studies have confirmed the effectiveness of kangaroo care and touching in relieving neonatal pain. Additionally, Zhou et al. reported that combining both methods significantly alleviated pain during ROP screening in preterm infants (25). However, clinical adoption remains limited by operational complexity, family member cooperation, and NICU management practices. Consequently, NNS has become the predominant clinical intervention.

NNS refers to using pacifiers or similar methods to stimulate the newborn's sucking action without ingestion of milk or sweeteners (8). Its primary mechanism involves producing analgesic effects by stimulating oral tactile receptors, thus raising the pain threshold and promoting 5-HT release (26). Additionally, sucking actions soothe premature infants, making NNS the most common non-pharmacological analgesia in neonatal units. Current guidelines (8, 27) recommend NNS for neonatal pain management. Nevertheless, the WHO's Baby-Friendly Hospital Initiative advocates minimizing pacifier use due to potential risks of nipple confusion and infections resulting from inadequate disinfection (28). Therefore, clinical implementation requires individualized protocols to balance associated risks and benefits.

### 3.2 Mechanism of auditory intervention

Auditory intervention alleviates pain by providing external auditory stimuli that inhibit or modulate central pain processing pathways. Currently, auditory interventions used in MSS include white noise, music, and maternal voice.

White noise refers to noise with a uniform power spectral density across the frequency spectrum (29). It promotes neurobehavioral maturation in neonates by modulating autonomic tone and inhibiting central pain signal transmission, thereby alleviating neonatal pain and associated symptoms (30). Common white noise sources include Orchard Enterprises’ album “Colic Baby: White Noise for Babies” (14) and Orhan Osman's album “Kolic” (31). Ren et al. explored the combined use of white noise and NNS during ROP screening in preterm infants. Their results indicated that while white noise and NNS individually reduced pain, their combination provided the greatest pain relief (14).

Music interventions offer a comfortable auditory environment for newborns, providing distraction. Additionally, sonic vibrations promote the synthesis and secretion of endorphins, relieving procedural pain and stabilizing physiological and behavioral states (31). Clinical guidelines predominantly recommend classical music and lullabies. Xu et al. provided lullabies beginning 5 min before and ending 5 min after procedures. Their results indicated that music therapy reduced pain scores, alleviated oxygen saturation declines, shortened recovery times for heart rate and oxygen saturation, and reduced crying duration in preterm infants. However, heart rate variability remained unaffected (32).

Maternal vocal stimulation involves using recordings of maternal heartbeats, speaking, singing, storytelling, or other maternal sounds in neonatal units to simulate intrauterine maternal vocal stimuli (8). Numerous studies recommend maternal vocal stimulation for pain relief in neonates ≥26 weeks of gestation (8, 31). However, maternal voice recordings are susceptible to external influences and vary significantly between individuals. Currently, no studies identify the most effective auditory stimulation method; comprehensive analyses should therefore consider factors including clinical department, individual neonates, and environmental conditions.

### 3.3 Mechanism of taste intervention

Taste interventions reduce neonatal pain through oral stimulation, triggering endogenous analgesic responses via taste receptors. Currently, MSS employs sweeteners (glucose, sucrose) and breast milk for taste interventions.

Oral sweeteners, administered using syringes, droppers, or pacifiers, activate receptors at the tongue's tip, triggering the release of endogenous opioids and thereby alleviating pain (8). Sweeteners have a pleasant taste and stable aftertaste, creating positive sensations for newborns and effectively reducing anxiety and other negative emotions during painful procedures. However, some researchers suggest repeated administration of sweeteners may pose risks to neonatal blood glucose levels, gastrointestinal function, and neurodevelopment (13). Studies differ regarding dosage, analgesic efficacy, and long-term consequences, with no universally accepted standard. Nonetheless, *the Expert Consensus on Neonatal Pain Assessment and Analgesia Management (2020 Edition)* recommends administering 0.2–0.5 ml/kg of 24% sucrose or 25% glucose solution two minutes before painful procedures (33). In a comparative analgesic efficacy study during ROP screening, researchers randomized neonates into breast milk, sucrose solution, and sterile water groups (34). The pain scores of the control group (sterile water) were significantly higher than those of the breast milk and sucrose groups, and the vital signs in the breast milk group recovered to baseline more rapidly. Thus, breast milk is recommended for clinical interventions.

Analgesic mechanisms of breast milk involve three primary pathways. First, breast milk contains abundant tryptophan, the precursor of melatonin and serotonin; melatonin produces analgesic effects by increasing β-endorphin levels (1). Second, breastfeeding constitutes a protective factor associated with reduced ROP risk (35). Third, breast milk aroma activates the neuropeptide system, enhances opioid activity, and produces analgesic effects (36), involving both taste and smell simultaneously. However, optimal dosing, administration modalities, and olfactory diffuser parameters remain unstandardized, necessitating further research on analgesic effectiveness.

## 4 Effect of MSS on ROP screening pain

Although single-sensory interventions are widely used for pain management during ROP screening in preterm infants, their analgesic efficacy remains controversial. Some studies have demonstrated the effectiveness of oral sweeteners (6–8), but contradictory findings exist. Nayak et al. randomized participants into three intervention groups (human milk, 10% glucose solution, sterile water). The results showed similar analgesic effects across the groups, indicating no significant pain reduction (37). Different results were reported by Dilli et al. (38), possibly due to differences in sweetener concentrations (10% glucose solution used by Nayak et al. vs. 24% sucrose solution used by Dilli et al.) and small sample sizes. Similarly, Li et al. demonstrated through meta-analysis that oral sugar solutions alone might not significantly relieve pain during ROP screening (39). Consequently, clinical studies on MSS have significantly increased in recent years.

Current multisensory approaches predominantly combine auditory-taste, auditory-tactile, or taste-tactile modalities. Hou et al. explored the analgesic effect of glucose combined with white noise on ROP screening pain. Subjects were divided into four groups: 25% glucose, white noise, combined intervention (25% glucose plus white noise), and control. Results showed significantly lower pain scores in the combined, glucose-only, and white-noise-only groups compared to the control group. Additionally, the combined group exhibited lower pain scores compared to either intervention alone, with statistically significant differences (39). Dilli et al. investigated the effect of NNS combined with 24% sucrose on ROP screening pain, dividing participants into two groups: NNS with 24% sucrose and NNS with sterile water. Results demonstrated a significantly reduced pain score in the sucrose combination group (38). Another study divided participants into four groups: control, white noise alone, NNS alone, and white noise combined with NNS. Findings indicated all interventions reduced pain compared to control, but the combined intervention had the greatest analgesic effect (14). Furthermore, some researchers have investigated kangaroo care combined with touching. Several international researchers have recommended non-pharmacological interventions for managing pain during ROP screening through meta-analyses and systematic reviews (6, 40). These analyses concluded that combined multisensory interventions provided synergistic effects superior to single-sensory methods. Given the superior analgesic efficacy, simplicity, and feasibility of combined white noise and NNS, this study recommends using this combination clinically.

However, MSS implementation requires medical staff with professional skills and experience. Additionally, preterm infants may respond differently to MSS. Implementation effectiveness varies with medical staff experience, education, and training, leading to inconsistent pain relief outcomes. Therefore, further research is needed to optimize combined intervention strategies, enhance synergistic effects, and reliably alleviate pain during ROP screening.

## 5 Summary

With the development of medical and health services, neonatal pain management has gradually received increased attention. Available evidence suggests pain can have short- and long-term adverse effects on neonates. Therefore, effective pain management is critical. MSS, through interventions targeting multiple sensory modalities, can alleviate pain and stabilize physiological indicators during ROP screening in preterm infants. Nevertheless, several limitations remain. First, long-term follow-up data are lacking, particularly regarding the effect of MSS on neurodevelopment. Second, intervention standardization remains insufficient, and no consensus exists on sensory stimulation parameters (sweetener dosage and concentration) or optimal intervention timing. Third, most studies are single-center with small sample sizes, providing limited evidence strength. Future research should include longitudinal studies collecting long-term neurodevelopmental data, establish evidence-based pain management protocols for ROP screening in preterm infants, and conduct large-sample, multicenter randomized controlled trials.
